# Comparison of single and dual growing rods in the treatment of early onset scoliosis: a meta-analysis

**DOI:** 10.1186/s13018-016-0413-y

**Published:** 2016-07-12

**Authors:** Gui-Jun Xu, Xin Fu, Peng Tian, Jian-xiong Ma, Xin-long Ma

**Affiliations:** Department of Orthopedics, Tianjin Hospital, Tianjin, 300211 People’s Republic of China; Biomechanics Labs of Orthopaedic Institute, Tianjin Hospital, No. 122 Munan Street, Hexi District, Tianjin, 300050 China

**Keywords:** Early onset scoliosis, Growing rod, Meta-analysis

## Abstract

**Background:**

The growing rod technique was applied in the treatment of early onset scoliosis (EOS) with promising outcomes and many complications at the same time. We reviewed data from literatures to compare the results of single growing rods with dual growing rods to achieve a clear understanding of this technique.

**Methods:**

PubMed, Embase, MEDLINE, ScienceDirect, CNKI, Wanfang Data, and CQVIP were searched electronically until March 2016 using “growing rod” and “early onset scoliosis” as major search terms. Also, we manually searched other relevant conference proceedings. Two reviewers independently finished methodological quality assessment, data extraction, and calculations.

**Results:**

Six retrospective trials were adopted in data analysis including 126 and 119 patients in the single and dual rod groups, respectively. Significantly better coronal correction rates were observed immediately after the initial operation (MD = −14.67; 95 % CI −20.97 to −8.37; *P* < 0.01; *I*^2^ = 0 %) and at the final follow-up (MD = −23.70; 95 % CI −45.87 to −1.52; *P* = 0.04; *I*^2^ = 82 %) in the dual rod group. Similarly, better lengthening of the T1–S1 height occurred in the dual rod group immediately after the initial operation (MD = −1.74; 95 % CI −2.62 to −0.85; *P* < 0.01; *I*^2^ = 0 %) and at final follow-up (MD = −3.8; 95 % CI −5.56 to −2.04; *P* < 0.001; *I*^2^ = 36 %). There were more complications about the implant in the single rod group, while wound problems were common in the other group.

**Conclusions:**

The data of the current meta-analysis showed advantages in the coronal correction rate and lengthening by dual growing rods with fewer implant-related complications and more wound complications.

## Background

Regardless of the etiology, early onset scoliosis (EOS) is a kind of scoliosis which attacks children before they are 5 years old [1]. Children who suffer from this type of scoliosis will experience rapid spine curve deterioration and cardiopulmonary disfunction later [2]. Therefore, timely treatment is needed to correct the scoliosis deformity and to offer patients more spine growth, less cardiopulmonary deficiency, and a better quality of life.

Cobb angle >35° or rib-vertebra angle difference >20° were the indicators for high possibility of progression. Therefore, immediate treatment should be conducted. Nonsurgical methods, such as casting and bracing, can be used. The results have shown, however, that these methods may hinder the development of the chest wall and then lead to cardiopulmonary disfunction [3]. Therefore, surgical intervention is suggested for these patients. Spinal fusion is not satisfactory for children 7 years of age or younger because it can impair respiratory function, cause cosmetic problems, and reduce the quality of life [4].

Compared with spinal fusion, fusionless surgery can correct the deformity and maintain spine balance, which can allow the spine to grow. The single distraction rod technique, initially described by Harrington and later modified by Moe et al., has been used to treat scoliosis [5, 6]. Despite having a better deformity correction rate, complication rates have ranged from 29 to 48 %, raising debates about its value in front of the non-satisfied benefit-risk ratio [7, 8]. To improve this technique with less complications, Akbarnia et al. [9] made some modifications on this technique with dual rods to gain more solid foundations. Studies later confirmed the advantages of the dual rod technique with safer and more effective outcomes, which can increase the stability of the whole implant structure.

Thompson et al. were the first ones to compare the advantages and limits between the single and dual growing rods. Their results showed that the structure with dual rods can provide stronger stability with better initial correction [10]. More recently, Akgul et al. [11] also reported better results from dual rod fixation. However, various rates of success and complications have been reported in the literature. Thus, we conducted this current meta-analysis to compare these two kinds of growing rod techniques in the treatment of EOS.

## Methods

### Search strategy

According to the Cochrane Collaboration guidelines, we searched PubMed (1966–2016.3), MEDLINE (1966–2016.3), Embase (1980–2016.3), ScienceDirect (1985–2016.3), CNKI (1985–2016.3), Wanfang Data (1985–2016.3), and CQVIP (1985–2016.3) to identify trials comparing these two techniques with the following terms “early onset scoliosis” and “growing rod” with the Boolean operators AND or OR. In addition, the reference lists of all included studies were manually searched to identify trials that may be missed. There was no restriction on language. The searching through titles and abstracts was assessed by two reviewers independently. Full-text articles were sometimes retrieved when a question existed based on the abstracts. We solved disagreements through discussion.

### Selection criteria

Inclusion criteria for trails were as follows: (1) infant patients diagnosed with EOS, (2) comparison of different kinds of growing rods, and (3) full-text articles with detail information. We excluded articles that applied other fusionless techniques, such as articles on the magnetically controlled growing rod (MCGR), for which we were unable to obtain the full texts, and papers without available information.

### Quality assessment

A quality assessment was conducted using the methodological index for non-randomized studies (MINORS) form for retrospective controlled trials [12]. The methodological quality score is from 0 to 24. Any disagreements were resolved by discussion.

### Data extraction

Information about study design, demographics, and outcomes for each group was extracted independently by two authors from the included articles. Contact to original authors for supplementary information was adopted when necessary.

### Data analysis and statistics methods

All the calculations were finished by RevMan 5.1 for Windows (Cochrane Collaboration, Oxford, UK). Standard chi-square test was used to test heterogeneity with *P* < 0.05 and an *I*^2^ value greater than 50 % considered as statistically significant [13]. A random-effect model was applied for pooled data with significant heterogeneity [14], while a fixed-effect model was used for the others. For continuous outcomes including C7–S1 height, coronal correction rate, mean difference (MD), and 95 % confidence intervals (CI) were calculated, while we calculated risk difference (RD) and 95 % CI.

## Results

### Literature search

**Fig. 1:**
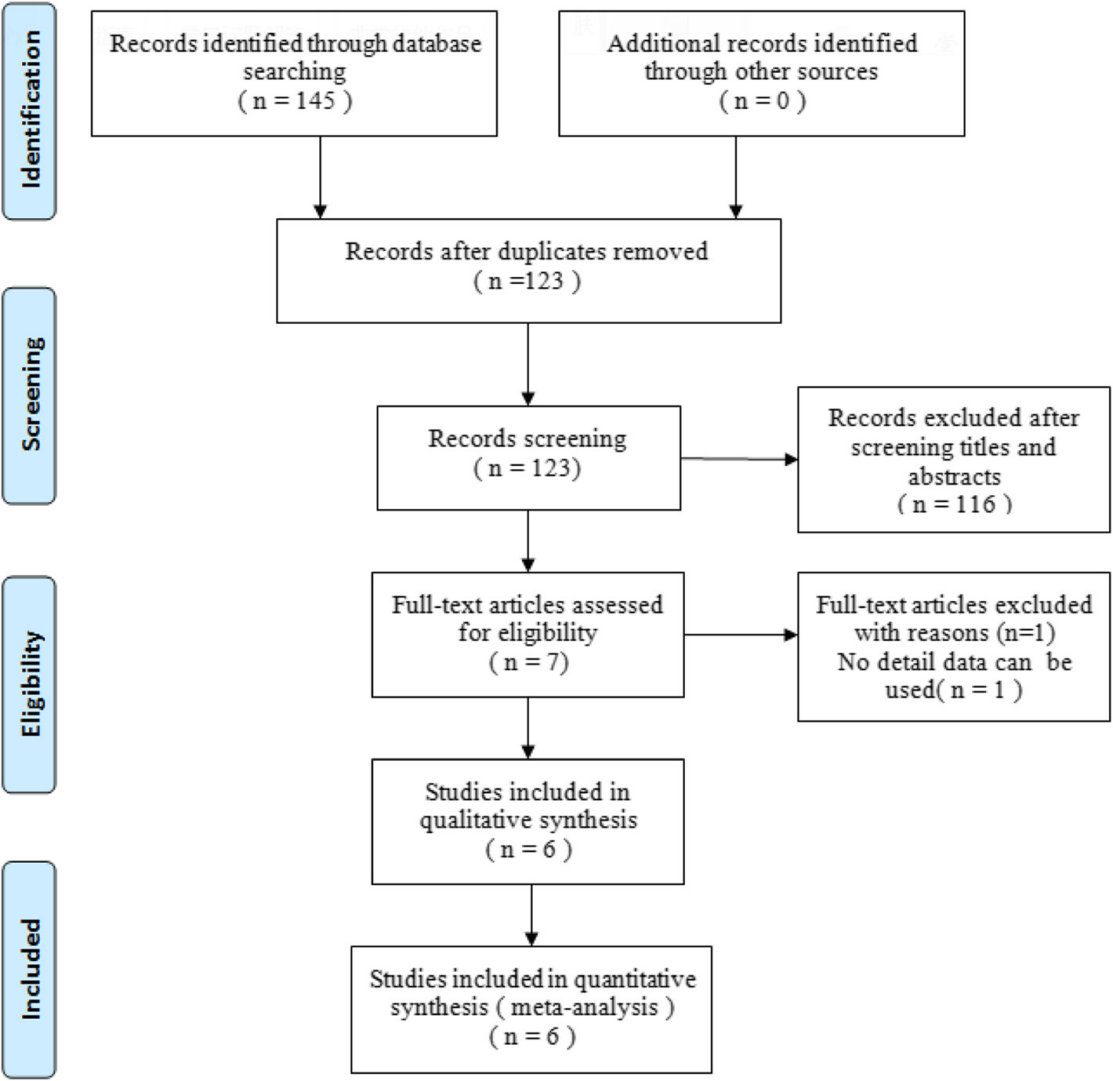
Flow chart of the study selection and inclusion process

The process of the literature selection and identification is shown in Fig. 1. According to the search strategy, 145 potential studies were identified, of which we excluded 139 studies based on the predefined criteria. Eventually, we identified six studies according to our search strategy [10, 11, 15–18].

#### Study characteristics

We extracted the available data from these articles without the data for those lost to follow-up. Excluding the patient who died 3 months post-operatively in the study of Sponseller et al. [15], these studies involved, respectively, 126 and 119 patients in the single and dual growing rod groups. We listed the characteristics of these included studies in Table 1.

**Table 1:** Characteristics of the included studies

Study	Groups	Patients (M/F)		Age at surgery (year)	Times	Internal (month)	Final fusion	Diagnoses	Follow-up (year or month)
Thompson, GH 2005 [10]	Single	21 (8/13)		8.3 ± 2.2 < 10 14, >10 7	2.9 ± 1.5	7.1 ± 4.9	21	Idiopathic 9, neuromuscular 7, congenital 1, other 4	7.6 ± 2.8 years
	Dual	7 (1/6)		7.0 ± 3.9 < 10 6, >10 1	6.1 ± 2.8	6.0 ± 0.4	7	Idiopathic 1, neuromuscular 1, congenital 1, other 4	6.0 ± 2.0 years
Sponseller, PD 2009 [15]	Single	2		8.4 ± 1.4	N	12.7 ± 6.9	5	Marfan syndrome 9	102.5 ± 1.5 months
	Dual	7		4.7 ± 2.3					82.6 ± 31.3 months
Bess, S 2010 [16]	Single	71 (41/30)		6.5	4 (0–11)	9.9 (2–24)	36	Idiopathic 40, neuromuscular 52, congenital 24, other 24	65.3 (24–166) months
	Dual	69 (30/39)		5.5	5 (2–13)	10.9 (5–33)	14		53.8 (24.7–126) months
Uzumcugil, O 2012 [18]	Single	11 (3/8)		7.5 ± 0.9	5.0 ± 0.6	N	N	Idiopathic 16, congenital 4	2.8 (2–4.8) years
	Dual	9 (2/7)		7.9 ± 1.3	4.1 ± 0.8	N	N		2.3(2–3.1) years
Zhao, Y 2012 [17]	Single	6 (2/4)		<10 5, >10 1	N	6–12	4	Neuromuscular 1, congenital 24	31.9 (12–89) months
	Dual	19 (6/13)		<10 18, >10 1			2		
Akgul, T 2014 [11]	Single	15	(9/14)	7.5 ± 2.2	2.1 ± 1.14	13 (2–28)	9	Idiopathic 11, neuromuscular 2, congenital 10	43.6 ± 23.2 months
	Dual	8					1		35.7 ± 14.6 months

### Quality assessment

Quality assessment scores ranged from 13 to 19. For all included studies, there were no descriptions about prospective calculations of the sample size or unbiased assessment of the study end points. With regard to the prospective collection of data, only two studies collected data prospectively [11, 17]. The study by Sponseller et al. [15] had the lowest quality score at 13. More details on the quality assessment are summarized in Table 2.

**Table 2:** Quality assessment score of the included studies

Quality assessment for non-randomized trials	Thompson, GH	Sponseller, PD	Bess, S	Uzumcugil, O	Zhao, Y	Akgul, T
A clearly stated aim	2	2	2	2	2	2
Inclusion of consecutive patients	2	2	2	1	1	1
Prospective data collection	0	0	1	0	2	2
End points appropriate to the aim of the study	2	0	2	2	2	2
Unbiased assessment of the study end point	0	0	0	0	0	0
A follow-up period appropriate to the aims of the study	2	2	2	2	2	2
Less than 5 % loss to follow-up	0	2	2	2	2	2
Prospective calculation of the sample size	0	0	0	0	0	0
An adequate control group	2	2	2	2	2	2
Contemporary groups	2	2	2	2	2	2
Baseline equivalence of groups	2	1	1	2	2	2
Adequate statistical analyses	2	0	2	2	2	2

### Outcomes of the meta-analysis

**Fig. 2:**
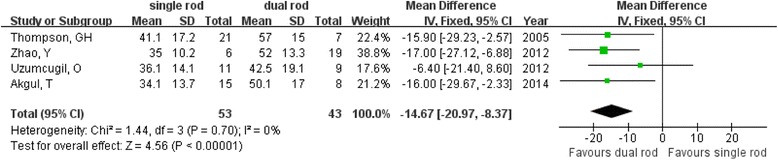
Forest plot showing initial coronal correction rate

**Fig. 3:**
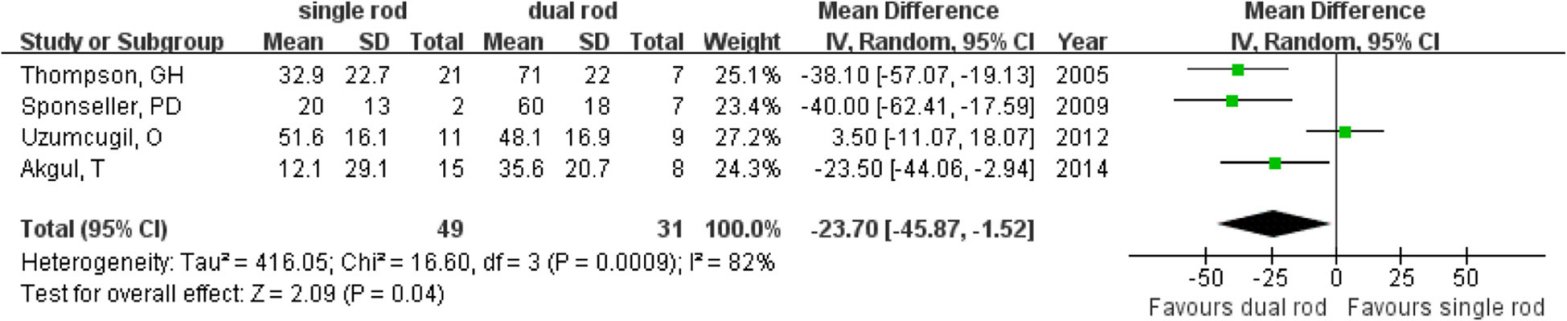
Forest plot showing long-term coronal correction rate

The dual growing rod group showed a significantly better initial change in coronal correction rate from before to immediately after the operation than did the single rod group (MD = −14.67; 95 % CI −20.97 to −8.37; *P* < 0.01; *I*^2^ = 0 %; Fig. 2) [10, 11, 17, 18]. Regarding the long-term correction rate, better results were also observed (MD = −23.70; 95 % CI −45.87 to −1.52; *P* = 0.04; *I*^2^ = 82 %; Fig. 3) in the dual growing rod group.

**Fig. 4:**
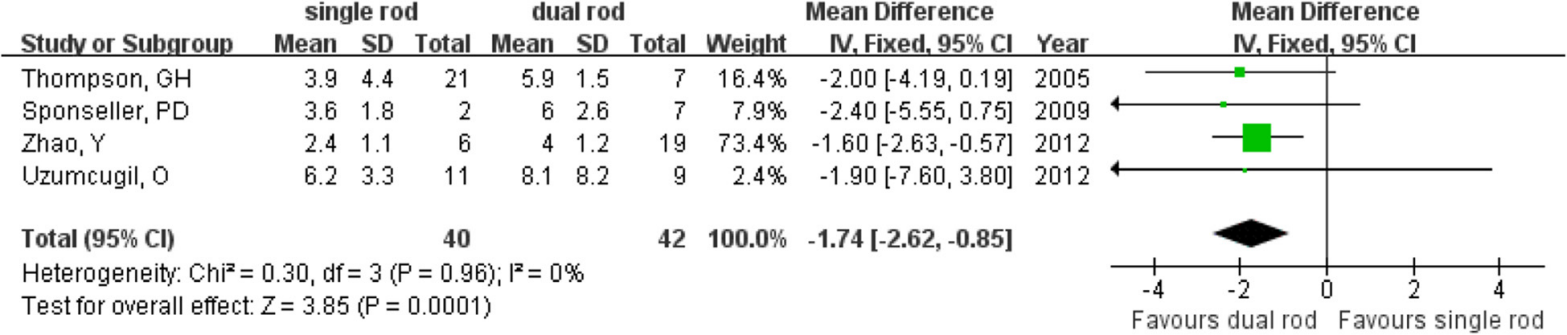
Forest plot showing initial lengthening

**Fig. 5:**
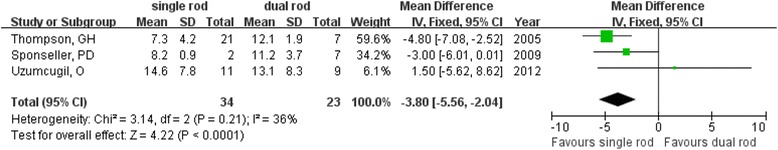
Forest plot showing long-term lengthening

The lengthening of the spine was measured as the C7–S1 height in the study by Zhao et al. [17] and as the T1–S1 height in three other studies [10, 15, 18]. We calculated the change in height to evaluate the initial effect of surgery on the spine based on all four studies and found much more lengthening in the dual growing rod group (MD = −1.74; 95 % CI −2.62 to −0.85; *P* < 0.01; *I*^2^ = 0 %; Fig. 4) than in the single growing rod group. This advantage can also be observed after long-term follow-up (MD = −3.8; 95 % CI −5.56 to −2.04; *P* < 0.001; *I*^2^ = 36 %; Fig. 5).

Complications were reported in all six included studies. To evaluate the incidence rate of complications, we calculated three different types of complications, including problems with the rod; problems with the hook, screw, and wire; and wound problems. More complications about the implants were noticed in the single rod group, including broken rods (RD = 0.17; 95 % CI 0.05 to 0.28; *P* = 0.005; *I*^2^ = 50 %) and problems with the hook, screw, and wire (RD = 0.14; 95 % CI 0.02 to 0.25; *P* = 0.02; *I*^2^ = 21 %). Wound complications, in contrast, happened more in the dual growing rod group (RD = −0.13; 95 % CI −0.23 to −0.04; *P* = 0.008; *I*^2^ = 16 %).

### Other outcomes

The included study by Zhao et al. [17] did not find a significant difference in blood loss during surgery, with 137 ± 84 ml in the single rod group versus 143 ± 66 ml in the dual rod group.

## Discussion

In the literature, the term “early onset” has been used to indicate scoliosis before 5 years old of all etiologies, while “late onset” indicates scoliosis after age five [19]. The distinction is made because of the difference in spine growth and cardiopulmonary compromise before and after age five [20]. “Early onset” was used in paper titles, but the included studies also admitted patients older than 5 years old and in some cases more than 10 years old. Therefore, our study showed the results of children generally younger than 10 years old. Based on the limited studies, better coronal correction rates and lengthening with fewer implant-related complications and more wound complications were noticed in the dual growing rod group.

The confirming power of a meta-analysis of non-randomized controlled trials (non-RCTs) is challenged mainly by inherent biases and differences in study designs. However, in many situations, RCTs are not feasible. A meta-analysis based on non-RCTs may provide a tool to understand and quantify sources of variability in results across studies [21]. In our study, only non-RCTs could be found, and risk existed because of the retrospective data collection, no blindness of evaluation, and difference in patients. Therefore, we urge caution and prudence when interpreting the results presented here.

Correcting the deformity and allowing the chance for the spine to grow are the major objectives of the growing rod technique. Because of the stronger biomechanics of the double instrument used in dual growing rod fixation, there was no doubt that a better correction could be achieved and maintained. In modern instrumentation systems, there is a trend to use the pedicle screws in segmental pedicle screw constructs or hybrid constructs [22], and a structure composed of four pedicle screws in two adjacent vertebral bodies can provide the strong pullout force [23].

As to the growth of children, frequency of lengthening is an important factor. Results from a previous study showed that more frequent lengthening based on dual rods can provided continued growth for the spine after the initial procedure with equaled or surpassed growth rate to normal spine [24]. With short lengthening interval (≤ every 6 months), patients can have a higher annual growth rate (1.8 vs. 1.0 cm) and greater scoliosis correction rate (79 vs. 48 %) than long lengthening interval (every 9–20 months) because of better spine control for dual growing rods. This may be why dual growing rods can achieve more improvement of the T1–S1 height. Different intervals of lengthening were used in the included studies, making it difficult to detect the actual effect of intervals on lengthening.

Although the growing rod provides good control of the spine, its value has been questioned because of many complications that can occur during the treatment process. A single rod cannot provide enough support for the spine when the patient is active. The included studies reported fewer complications related to implants in the dual growing rod group, as did the meta-analysis. There may also have been benefits from the stronger biomechanics of dual rod fixation with dissipated mechanical stress compared with a single rod. Frequent lengthening procedures with many surgeries increase the possibility of wound problems. Younger children’s body structure, including less soft tissue coverage, may also contribute to more wound problems with dual growing rods. A new method, called magnetically controlled growing rods, involves fewer surgeries and is a promising method for reducing wound complications [25].

Some limitations must be clarified about our present work. First, the results provided in this article come from six retrospective studies. The convincing power of the meta-analysis is limited by the lack of high-quality RCTs. We will keep our eyes on new studies about the growing rods and draw a clearer conclusion based on studies with high quality, especially RCTs. Second, based on the limitations of the included studies, we calculated the spine growth during treatment from T1 to S1 and included the C7–S1 growth from the study by Zhao et al. [17], which brings heterogeneity to the results. Another weakness is that we only calculated the coronal correction rate. For comprehensive understanding of the effects of the growing rod, more radiographic parameters should be evaluated, such as different types of scoliosis, kyphosis, and lordosis. It has been reported that significantly more complications showed in patients with kyphosis angle ≥40° who received growing rod surgery [26]. In a study with 25 patients, on the contrary, Caniklioglu et al. [27] found that the single rod technique can significantly improve and maintain shoulder balance compared with the dual rod technique.

## Conclusions

The results of this review showed that the dual growing rod technique can achieve a better coronal correction rate and lengthening with fewer implant-related complications and more wound complications. High-quality, randomized controlled trials are required to confirm our findings.

## Abbreviations

CI, confidence interval; EOS, early onset scoliosis; MCGR, magnetically controlled growing rod; MD, mean difference; RCT, randomized controlled trial
